# Interplay Between KSHV and the Host DNA Damage Response

**DOI:** 10.3389/fcimb.2020.604351

**Published:** 2020-12-09

**Authors:** Eriko Ohsaki, Keiji Ueda

**Affiliations:** Division of Virology, Department of Microbiology and Immunology, Osaka University Graduate School of Medicine, Suita, Japan

**Keywords:** Kaposi’s sarcoma-associated herpesvirus, DNA damage response, DNA repair, cell cycle, latency, lytic replication, KSHV, DDR

## Abstract

Interactions between viruses and cellular factors are essential for viral replication or host defense. The DNA damage response (DDR) orchestrates a molecular network of cellular mechanisms that integrates cell cycle regulation and DNA repair or apoptosis. Numerous studies have revealed that the DDR is activated by virus infection, aberrant DNA structures generated by viral DNA replication, or the integration of retroviruses. Although the DDR is an essential function for maintaining the genomic integrity of cells, viruses may utilize this mechanism to build a convenient environment for themselves, and the resulting perturbation of the DDR has been shown to increase the risk of tumorigenesis. There have been many studies investigating the roles of the DDR in oncogenic viruses such as Epstein-Barr virus (EBV), human papillomavirus (HPV), hepatitis B virus (HBV), human T-cell leukemia virus type 1 (HTLV-1), and Kaposi’s sarcoma-associated herpesvirus (KSHV). This review summarizes current knowledge on the roles of DDR in the KSHV lifecycle.

## Introduction

For the survival of organisms, the faithful transmission of genetic information from a parent cell to its daughter cells is essential. Such accurate transmission requires not only mechanisms for the faithful replication of DNA and segregation of chromosomes, but also mechanisms to prevent spontaneous and/or exogenously induced DNA damages. To accomplish all these goals, cells have monitoring systems that survey aberrant chromosomal structures. After sensing DNA damage, a DNA damage checkpoint coordinates with the cell-cycle regulation and repair systems. In response to DNA damage, the DNA damage response (DDR) controls cell cycle arrest to allow enough time for repair. When DNA damage is too severe to rescue, the apoptosis pathway is activated by the DDR.

KSHV is classified as a member of the Gammaherpesvirinae subfamily, which also includes Kaposi’s sarcoma and lymphoproliferative disorders such as primary effusion lymphoma (PEL) and multicentric Castleman’s disease (MCD), and KSHV is often associated with HIV infection (Cesarman and Knowles, 1995; Soulier et al., 1995). KSHV has two distinct lifecycles, a latent phase and lytic replication phase. During latency, the viral genome is maintained with limited gene expression in host cells (Sarid et al., 1998; Fakhari and Dittmer, 2002; Lieberman, 2013; Campbell et al., 2020). When latency is disrupted, the virus shifts to a lytic phase in which infectious progeny virions are produced (Sun et al., 1998; Purushothaman et al., 2015; Aneja and Yuan, 2017).

Affinity purification of DNA-binding proteins first demonstrated that several DDR proteins such as poly (ADP-ribose) polymerase 1 (PARP1) and MutS homolog 2/3/6 (MSH2/3/6) bind to the terminal repeat (TR) region (Ohsaki et al., 2004), and that MSH2/6, PARP1, DNA-dependent protein kinase (DNA-PK), and Ku70/80 bind to lytic DNA replication origins (ori-Lyt) (Wang et al., 2008). Recently, many studies have reported that DDR proteins are upregulated by viral replication and involved in the KSHV lifecycle, as described in a later section.

Viral infection causes global disruption of nuclear architecture and chromosomal aberration, and DDR is activated as a potent antiviral defense (Fortunato and Spector, 2003). In addition, structures of the viral genome, including linear double stranded DNA (dsDNA) (herpesviruses, adenoviruses), circular dsDNA (polyomaviruses, papillomaviruses), and RNA genomes which are reverse transcribed to linear dsDNA (retroviruses), can be recognized by DDR sensor proteins (Everett, 2006; Kerur et al., 2011; Lilley et al., 2011; Barber, 2014; Hau and Tsao, 2017; Kleinberger, 2020). On the other hand, viruses take advantage of the DDR pathway to modulate the cell cycle and hijack cellular proteins to support viral replication (Everett, 2006; Lilley et al., 2010; Weitzman and Fradet-Turcotte, 2018). Since DDR signaling pathways induce cell cycle arrest or apoptosis, which are negative effects on virus production, viruses have developed suppressive strategies against the DDR. Over the past two decades, numerous studies have reported the interactions between DDR signaling pathways and human tumor viruses including HPV, HTLV-1, HBV, HCV, EBV, and KSHV (Weitzman and Fradet-Turcotte, 2018).

Deregulation of DDRs because of the competition among such virus-host defense systems increases the risk of tumorigenesis. This is because various kinds of cell signaling networks maintaining homeostasis are perturbed. Therefore, an improved understanding of these relationships between viruses and DDR systems will help us to develop strategies for anti-tumorigenic and anti-viral therapies. In the first section of this review we summarize DDR signaling pathways, and in the second section we focus on the relationships between KSHV and DDR.

## DNA Damage Response

**Figure 1:**
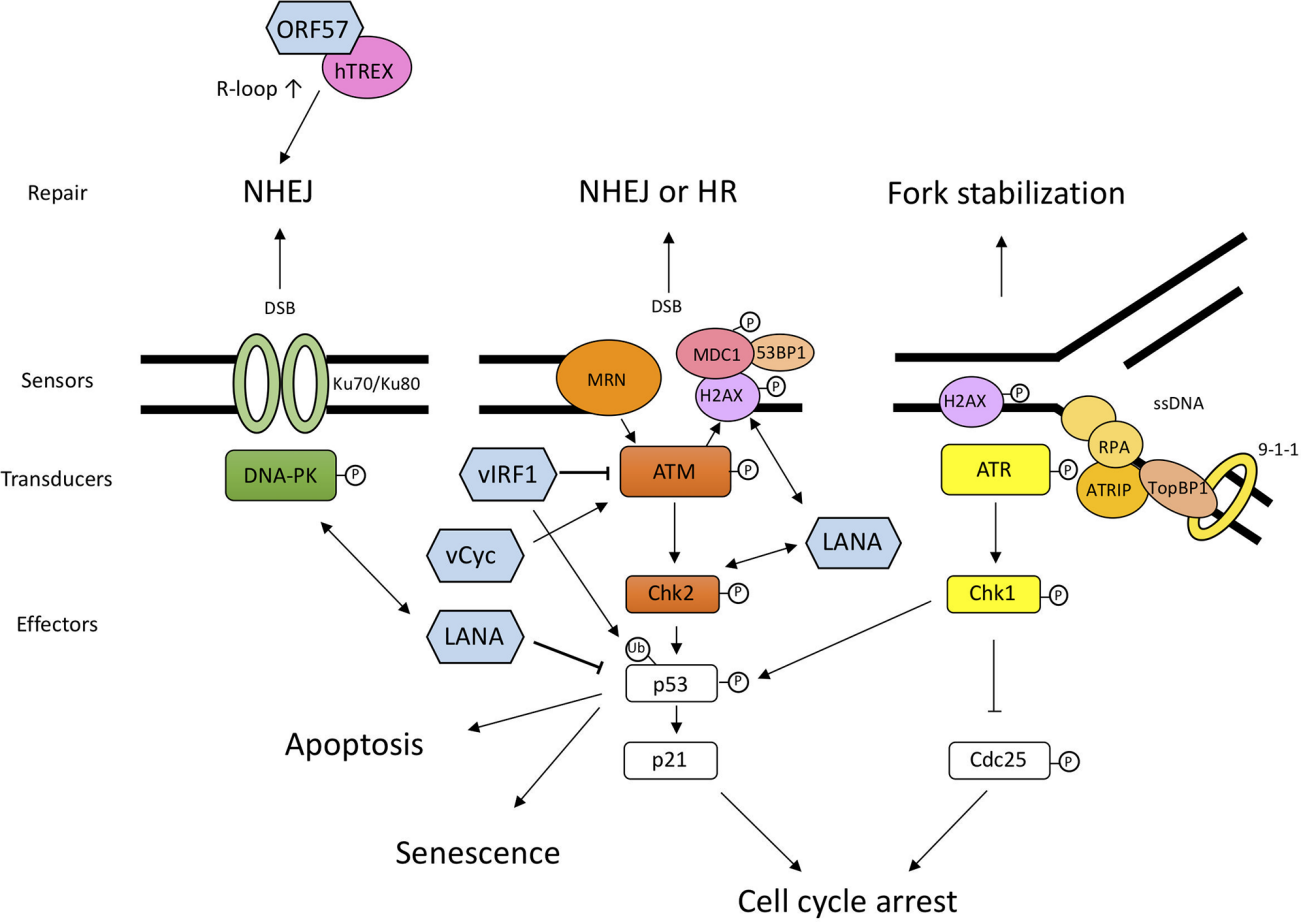
Schematic diagram of DDR pathways and the interplay between DDR and KSHV proteins. DNA damage sensors recognize the aberrant DNA structure and activate transducers, such as DNA-PK, ATM, and ATR. These transducers activate downstream effectors related to cell cycle arrest and DNA repair. When the DNA damage is too severe, cells undergo permanent cell-cycle arrest, senescence, or cell death. The viral proteins shown in this figure are not exhaustive but include the proteins mentioned in this article. LANA and v-cyclin lead to the activation of sensors and transducers *via* direct or indirect interaction. vIRF1 interacts with ATM and downregulates its kinase activity and ubiquitinates p53 to degrade it. ORF57 expression induces several NHEJ proteins and interacts with hTREX, and consequently transcribed mRNA becomes unstable and forms R-loops, which leads to genomic instability. The double-headed arrow shows the interaction between viral protein and DDR protein. DNA-PK, DNA-dependent protein kinase; ATM, Ataxia telangiectasia mutated; ATR, ATM and Rad3-related; MRN, Mre11-Rad50-NBS1 complex; Chk1, Checkpoint kinase 1; Chk2, Checkpoint kinase 2; MDC1, mediator of DNA damage checkpoint protein 1; ATRIP, ATR-interacting protein; RPA (replication protein A); TopBP1, DNA topoisomerase II binding protein 1.

DDR consists of DNA damage sensors followed by transducers, and effectors (**Figure 1**). All the DDR pathways including cell cycle checkpoints and DNA repair pathways are completed by proper signaling from sensors to transducers and to effectors.

### DNA Damage Sensors and Transducers

#### PIKKs: Transducers of DNA Damage Response

The cellular responses to DNA damage are mainly controlled by three phosphatidylinositol 3-kinase-like kinases (PIKKs): ATM (Ataxia telangiectasia mutated), ATR (ATM and Rad3-related), and DNA-PK (Cimprich and Cortez, 2008; Davis et al., 2014; Blackford and Jackson, 2017; Menolfi and Zha, 2020), which act as DNA damage transducers. ATM and DNA-PK are principally activated in response to double-strand breaks (DSBs). In contrast, ATR is activated by single-stranded DNA (ssDNA) during the S-phase to regulate the firing of replication origins and the stalled replication forks.

#### DNA Damage Sensing and Signaling

DSBs are recognized by the MRN complex, which is composed of Mre11, Rad50, and NBS1 and activates ATM (Lavin et al., 2015). MRN mediates cross-talk among the repair and checkpoint machinery. ATM phosphorylates downstream molecules, such as H2AX, a variant of the histone H2A protein family, and Chk2 (Checkpoint kinase 2) (Burma et al., 2001; Bartek and Lukas, 2003). Phosphorylated H2AX (γH2AX) is generated in chromatin near DSBs and recruits critical adaptor proteins such as MDC1 (mediator of DNA damage checkpoint protein 1) and 53BP1 (p53 binding protein 1) (Stewart et al., 2003; Xie et al., 2008; Kilic et al., 2019). Activated ATR and ATM phosphorylate the downstream targets Chk1 (Checkpoint kinase 1) and Chk2, which are key effectors in DDR (Bartek and Lukas, 2003; Smith et al., 2020).

Ku70/Ku80 are other sensor proteins recognizing DSBs and recruit the DNA-PK, and their main role is to facilitate non-homologous end joining (NHEJ), which is one of the DNA repair systems, as discussed in a later section (Jette and Lees-Miller, 2015; Chang et al., 2017). Following the sensing of DNA lesions, phosphorylation of transducer and effector molecules by PIKKs induces cell cycle arrest, DNA repair, and/or apoptosis or senescence.

### Cell Cycle Checkpoints and DNA Repair Pathways

Activated Chk1 and Chk2 phosphorylate downstream targets such as Cdc25 and p53 followed by degradation of Cdc25 and cell cycle arrest (Donzelli and Draetta, 2003; Liu et al., 2020) or by activation of the p53-mediated signaling pathway for DNA repair, cell cycle arrest, and apoptosis (Bartek and Lukas, 2003; Williams and Schumacher, 2016). p21, the downstream target of p53, induces cell cycle arrest through inhibition of the cyclin E/Cdk2 complex (Waldman et al., 1995; Planas-Silva and Weinberg, 1997; Gire and Dulić, 2015).

PIKKs have roles in the recruitment of repair machineries *via* the phosphorylation of downstream elements. For DSB repair, two major pathways are used: NHEJ and homologous recombination (HR). The NHEJ pathway has largely four steps: recognition, resection, polymerization, and ligation of the DNA ends (Chang et al., 2017). In the NHEJ, Ku70/80 primarily recognizes DSBs, and DNA-PK is recruited and activated by Ku-bound DSB ends to promote NHEJ.

DSBs are also recognized by the MRN complex (Carson, 2003), which promotes ATM activation and resection of DSB ends to generate ssDNA overhangs. While NHEJ is active throughout interphase, HR is active only in the S and G2 phases, because a homologous chromosome is available in the S and G2 phases as a template for DNA repair. The relationships between HR proteins and viruses have been reported in EBV (Kudoh et al., 2009), HPV (Gillespie et al., 2012; Park et al., 2014), and HTLV-1 (Belgnaoui et al., 2010), but not in KSHV. Additional investigations will be needed to expand our knowledge of the relationships between KSHV and HR.

The mismatch repair system improves DNA replication fidelity by degrading an error-containing region of the newly synthesized strand and providing a chance for the DNA polymerase to correct errors (Li, 2008; Fishel, 2015; Gupta, 2019). MSH2, MSH3, and MSH6 participate in mismatch repair as heterodimers, i.e., MSH2/MSH6 or MSH2/MSH3. While MSH2/6 recognizes base pair mismatches and small insertion/deletions, MSH2/MSH3 recognizes various DNA mismatches, including DNA loops ranging from 1 to 14 nucleotides as well as longer insertion/deletion mismatches (Jiricny, 2006). Several studies have suggested that the mismatch repair pathway is involved in viral replication not only in KSHV (Ohsaki et al., 2004; Wang et al., 2008; Cha et al., 2010), but also in EBV (Daikoku et al., 2006), as described in a later section.

Poly (ADP-ribose) polymerase 1 (PARP1) is an ADP-ribosylating enzyme and a multifunctional nuclear enzyme that affects various aspects of cellular homeostasis, such as DNA repair, cell proliferation, apoptosis, and inflammation. PARP1 is another important SSB- and DSB-signaling protein and modifies both target proteins and PARP1 itself by poly (ADP) ribosylation (Ray Chaudhuri and Nussenzweig, 2017). PARP1 has a pivotal role in DNA repair and is involved in various repair pathways, such as single-strand break repair (SSBR) (Leppard et al., 2003; Fisher et al., 2007), base excision repair (BER) (Masson et al., 1998; Dantzer et al., 2000; Lavrik et al., 2001; El-Khamisy, 2003; Ronson et al., 2018), nucleotide excision repair (NER) (Pines et al., 2012; Robu et al., 2017), NHEJ (Wang et al., 2006; Mansour et al., 2010; Cheng et al., 2011; Spagnolo et al., 2012; Luijsterburg et al., 2016), and HR (Hochegger et al., 2006; Hu et al., 2014).

## Interplay Between KSHV and DDR

### Roles of the DDR in *De Novo* Infection and Latent Infection

During the latent phase, the KSHV genome persists in the host nucleus as a double-stranded circular DNA—i.e., as an extra-chromosomal viral genome (episome)—and a very limited set of viral genes such as LANA (ORF73), vFLIP (ORF71), v-cyclin (ORF72), Kaposin (ORF K12), vIRF3 (LANA2), and 12 miRNAs are expressed (Dittmer et al., 1998; Sun et al., 1999; Parravicini et al., 2000; Cai et al., 2005; Pfeffer et al., 2005; Samols et al., 2005; Lieberman, 2013; Qin et al., 2017).

Like the proteins of other viruses, the KSHV proteins interact with DDR components and activate or prevent the signaling response (**Figure 1** and **Table 1**). *De novo* infection of KSHV in human PBMCs or primary endothelial cells upregulates the level of γH2AX, which is the phosphorylated form of H2AX (Jha et al., 2013; Singh et al., 2014). γH2AX interacts with LANA and contributes to LANA-mediated episome maintenance (Jha et al., 2013). It has been reported that H2AX knockdown reduces the expression of LANA and viral genome copies, suggesting that γH2AX has a role in latent gene expression and establishment of KSHV latency (Singh et al., 2014). In the same manner as phosphorylated H2AX, phosphorylated ATM was induced within 30 min post infection, and the inhibition of ATM activity caused a reduction of LANA expression, while knockdown of Chk1 and Chk2 did not affect LANA expression (Singh et al., 2014). These results suggest that selective activation of the DDR pathway is critical for the initial stages of KSHV infection and establishment of viral latency.

**Table 1:** List of DDR-KSHV interaction during *de novo* infection, latency, and lytic reactivation and its effects.

	Cellular DDR proteins	Viral components	Effects	Refs.
**De novo infection**	γH2AX	LANA	Episome persistence	Jha et al., 2013
	γH2AX, ATM	?	Establishment of latency	Singh et al., 2014
**Latent**	PARP1	TR, LANA	Negative for virus maintenance	Ohsaki et al., 2004
	ATM, Chk2, γH2AX, p53	v-cyclin	Oncogenic	Koopal et al., 2007
	DNA-PK, Ku70/80	LANA	Negative for virus maintenance	Cha et al., 2010
	Chk2	LANA	Protection from G2/M cell cycle arrest	Kumar et al., 2014
	GADD45B	miRNA-K9	Anti-apoptotic, Protection from cell cycle arrest	Liu et al., 2017
	MRN	cytoplasmic LANA (LANA⊿N)	Modulation of an innate immune signaling pathway	Mariggiò et al., 2017
**Lytic**	PARP1	RTA	Abortive lytic replication	Gwack et al., 2003
	ATM, p53	vIRF1	Downregulation of DDR for viral replication	Shin et al., 2006
	PARP1	ori-Lyt	Positive for lytic DNA replication	Wang et al., 2008
	Ku70/80, DNA-PK, MSH2/6	ori-Lyt	Supportive for lytic DNA replication	Wang et al., 2008
	TIP60	ORF36	Positive for lytic gene expression	Li et al., 2011
	γH2AX, Mre11, Rad50, Ku70/80, DNA-PK, PARP1, XRCC1, DNA ligase 3	ORF57	Genomic instability	Jackson et al., 2014
	RPA, Mre11	lytic replication foci	Positive for viral DNA synthesis	Hollingworth et al., 2015
	DNA-PK, Ku80	lytic replication compartments	Negative for viral DNA replication	Hollingworth et al., 2017
	MRN	lytic replication compartments	Positive for viral DNA replication	Hollingworth et al., 2017

DDR proteins are equally distributed in the nucleus, but recent works have suggested that cytoplasmic DDR proteins sensor the cytoplasmic exogenous DNA and activate the innate immune signaling (Roth et al., 2014). Mariggio et al. demonstrated that the cytoplasmic LANA recruits the MRN complex in the cytoplasm of KSHV-infected B cells to inhibit NF-κB activation and blocks the role of innate immune sensors of cytoplasmic DNA (Mariggiò et al., 2017).

The cell cycle profiles of KSHV-positive cells suggest that LANA inhibits nocodazole-induced G2/M arrest (Kumar et al., 2014). In the same study, Kumar et al. (2014) reported that LANA interacts with Chk2 through the serine rich N-terminal domain of Chk2, and that downregulation of Chk2 expression promotes G2/M arrest in KSHV-positive BC3 cells. These results suggested that LANA interacts with Chk2 to escape from the G2/M cell cycle arrest due to the ATM/ATR signaling pathway.

Some of the DDR proteins play negative roles in latent DNA replication (Koopal et al., 2007; Cha et al., 2010). Using a proteomics approach, Cha et al. (2010) identified DNA-PK, Ku70, and Ku80 as LANA-binding proteins. They further showed that LANA is phosphorylated by DNA-PK/Ku and reduces transient DNA replication. Finally, they reported that overexpression of Ku70 downregulates transient DNA replication, suggesting that the DNA-PK/Ku complex binds with LANA and negatively regulates latent replication (Cha et al., 2010).

KSHV v-cyclin induces replicative stress in EA.hy926—which is a HUVEC-epithelial A549 hybrid cell line and has been used as an endothelial cell model—and also induces DDR and senescence by activating γH2AX, ATM, Chk2, p53, and p21 (Koopal et al., 2007). This v-cyclin-induced DDR is dependent on CDK6, a catalytic subunit of the v-cyclin. From this study, the DDR response appears to be activated by host defense mechanisms.

In addition, a recent study suggested that KSHV miRNAs target GADD45B to protect infected cells from cell cycle arrest and apoptosis (Liu et al., 2017). KSHV infection in primary endothelial cells causes repression of growth arrest DNA damage-inducible gene 45 (GADD45B). This study also demonstrated that KSHV miRNA-K9 inhibits the expression of GADD45B induced by a p53 activator, Nutlin-3, and suggested that KSHV miRNAs play essential roles in protecting cells from the DDR-induced cell cycle arrest and apoptosis.

### Roles of the DDR in Lytic Replication

Murine γ-herpesvirus 68 (MHV68) ORF36, which is a conserved serine/threonine protein kinase in Herpesviridae and is similar to the cellular kinase cdk2 (Romaker et al., 2006), phosphorylates H2AX (Tarakanova et al., 2007) during lytic replication. This ORF36-mediated H2AX phosphorylation is dependent on ATM activity, and is critical for viral replication, suggesting that the association between viral kinase and cellular DDR proteins synergistically supports viral replication. Another study demonstrated that ORF36 phosphorylates histone acetyltransferase TIP60, an upstream regulator of the DDR pathway, and promotes lytic gene expression (Li et al., 2011).

MDM2, an E3 ubiquitin ligase and a repressor of p53, has a negative role in viral reactivation (Balistreri et al., 2016). Lytic reactivation induces a p53 response in PEL cell lines and arrests the cells at G2 phase, which enables efficient lytic replication, as a positive effect of DDR on viral replication.

A proteomics analysis based on SILAC (stable isotope labelling by amino acids in cell culture) identified a large number of NHEJ proteins, including Rad50, Mre11, DNA-PK, Ku70, Ku80, PARP1, XRCC1 (X-ray repair cross-complementing protein 1), and DNA ligase3, that are enriched upon the expression of KSHV ORF57, a viral early protein and post-transcriptional regulator of gene expression (Jackson et al., 2014). In addition, as a consequence of the interaction between ORF57 and hTREX (human transcription and export complex), which is an mRNA export complex, the newly transcribed mRNA becomes unstable and forms R-loops, leading to genomic instability.

Affinity purification and mass spectrometry assays have identified MSH2, MSH3, MSH6, PARP1, DNA-PK, and Ku70/Ku80 as TR-binding proteins (Ohsaki et al., 2004), as ori-Lyt-binding proteins (Wang et al., 2008), and as LANA-binding proteins (Cha et al., 2010). Lytic reactivation in RTA-inducible BCBL1 and KSHV-infected endothelial cells causes DDR activation through the phosphorylation of H2AX, ATM, and DNA-PK and modulates cell cycle progression (Hollingworth et al., 2015). RPA (replication protein A), which is a single-stranded DNA-binding protein, and Mre11 accumulate at viral replication foci, suggesting that DDR proteins contribute to viral DNA replication. These studies suggested that NHEJ proteins such as the MSH2/6 heterodimer and DNA-PK/Ku70/Ku80 heterotrimer are recruited to latent/lytic replication origins and have some roles in the formation of a replication initiation complex.

Another study also demonstrated that the Ku70/Ku80 heterodimer and the MRN complex are recruited to viral replication compartments (RCs) during lytic replication, and the activation of ATM kinase promotes viral replication (Hollingworth et al., 2017). On the other hand, the other DDR proteins, such as γH2AX, MDC1, and 53BP1, localize on the periphery of viral RCs. In addition, knockdown or inhibition of NHEJ proteins such as Ku80 and DNA-PK enhances viral replication, suggesting a negative effect of the NHEJ pathway on viral replication (Hollingworth et al., 2017). Thus, it still remains to be clarified whether such DNA repair components have positive or negative roles in KSHV viral replication. Further work is necessary to elucidate how DNA repair components function in viral lytic replication.

PARP1 has essential roles in posttranslational modification of a large number of target proteins related to various kinds of cellular events and acts as a multifunctional enzyme. A previous study reported that PARP1 inhibits viral transcription through RTA ribosylation and leads to abortive lytic replication (Gwack et al., 2003). PARP1 directly binds to the TR and ribosylates LANA to modulate viral replication in latency (Ohsaki et al., 2004; Cha et al., 2010). From these studies, PARP1 has negative roles in latent viral DNA replication and in lytic gene expression. On the other hand, another study reported that PARP1 plays a positive role in the early stage of viral DNA replication in lytic reactivation (Wang et al., 2008).

Although the biological significance of DDR activation in KSHV lytic/latent replication is not clear, the activation of upstream signaling of DDR seems to benefit the viral replication. In contrast, some viral proteins directly interact with the downstream signaling components to prevent effectors from suppressing virus replication. Downstream of ATM pathway are inactivated in the late stage of lytic reactivation through the vIRF1 (viral interferon regulatory factor 1)-mediated pathway (Shin et al., 2006). vIRF1 interacts with ATM and downregulates ATM kinase activity and the p53 protein level (Shin et al., 2006).

## Conclusions

**Figure 2:**
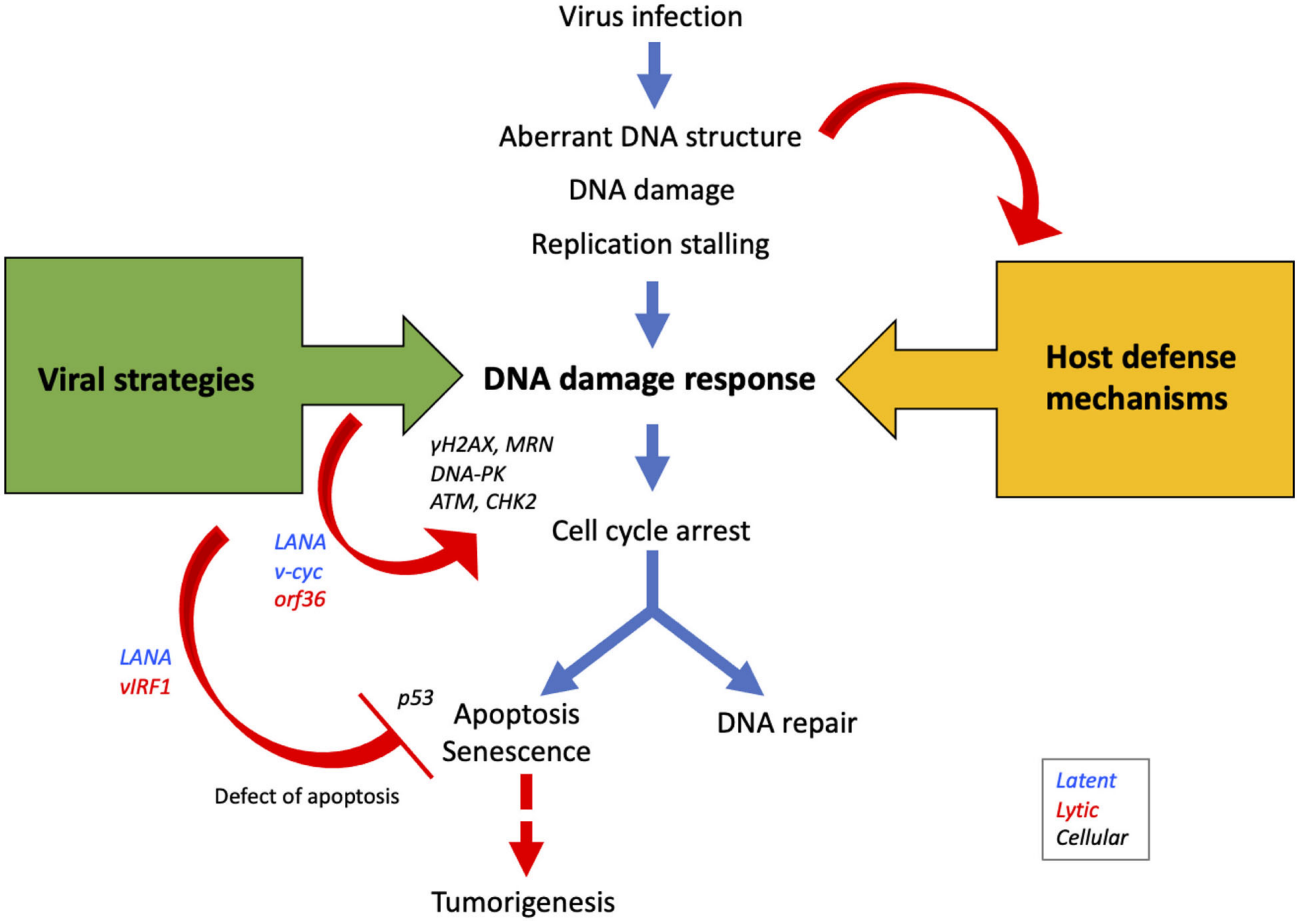
Viral proteins manipulate DDR signaling pathways to promote viral propagation. During infection, aberrant DNA structures or DNA damages caused by virus replication are recognized by DDR sensors/transducers as host defense mechanisms. On the other hand, viral proteins such as LANA, v-cyc, and ORF36 activate DDR proteins to modulate the cell cycle in the S-phase to promote viral replication. Since apoptosis decreases the opportunity for viral propagation, many viral proteins such as LANA, vIRF1, and vFLIP inhibit apoptosis and promote cell survival. The activation of the apoptotic pathway following the DDR occurs *via* p53, but because viral proteins deregulate this pathway, the risk of tumorigenesis is increased.

Over the past decades, many studies have elucidated the relationships between KSHV and the DDR signaling pathway. The roles of the DDR in viral replication depend on the type of infection, the structure of the viral genome formed by replication, the cell type, and the cell cycle stage. Aberrant DNA structures and signaling in the course of viral genome replication lead to the DDR pathway as a host defense response. On the other hand, viruses have developed strategies to hijack the DDR signaling pathway for their survival (**Figure 2**). A number of studies introduced in this article suggest that activation of the upstream pathway of the DDR—which includes DNA damage sensors and transducers—contributes to the modulation of both cell cycle progression and viral replication, whereas the downstream signaling pathways, such as the apoptosis pathway, are unfavorable for viruses. Accordingly, KSHV probably has developed strategies to negate a part of the DDR pathway.

Deregulation of the DDR pathway caused by such viral strategies increases the risk of tumorigenesis. More specifically, viruses affect cell cycle regulation to drive viral replication and manipulate the DDR pathway, and the resulting damage to the cellular repair system, increase in mutations, and resistance to apoptosis causes genomic instability and finally promotes tumorigenesis. An improved understanding of the battles between viruses and the DDR will lead to new therapeutic options for controlling viral replication and oncogenesis.

## Author Contributions

EO contributed to the design of the manuscript. EO and KU contributed to the concept of the manuscript. KU proofread and modified the manuscript. All authors contributed to the article and approved the submitted version.

## Conflict of Interest

The authors declare that the research was conducted in the absence of any commercial or financial relationships that could be construed as a potential conflict of interest.
